# Expression of tropomyosin in relation to myofibrillogenesis in axolotl hearts

**DOI:** 10.1186/2050-490X-1-8

**Published:** 2013-12-04

**Authors:** Robert W Zajdel, Matthew D McLean, Syamalima Dube, Dipak K Dube

**Affiliations:** Department of Medicine, SUNY Upstate Medical University, 750 East Adams Street, Syracuse, NY 13210 USA; Department of Cell and Developmental Biology, SUNY Upstate Medical University, 750 East Adams Street, Syracuse, NY 13210 USA

**Keywords:** *Ambystoma mexicanum*, Cardiac lethal mutation, Non-beating ventricle, Conus, Ectopic expression, Translational repression

## Abstract

The anatomy, function and embryonic development of the heart have been of interest to clinicians and researchers alike for centuries. A beating heart is one of the key criteria in defining life or death in humans. An understanding of the multitude of genetic and functional elements that interplay to form such a complex organ is slowly evolving with new genetic, molecular and experimental techniques. Despite the need for ever more complex molecular techniques some of our biggest leaps in knowledge come from nature itself through observations of mutations that create natural defects in function. Such a natural mutation is found in the Mexican axolotl, *Ambystoma mexicanum*. It is a facultative neotenous salamander well studied for its ability to regenerate severed limbs and tail. Interestingly it also well suited to studying segmental heart development and differential sarcomere protein expression due to a naturally occurring mendelian recessive mutation in cardiac mutant gene “c”. The resultant mutants are identified by their failure to beat and can be studied for extended periods before they finally die due to lack of circulation. Studies have shown a differential expression of tropomyosin between the conus and the ventricle indicating two different cardiac segments. Tropomyosin protein, but not its transcript have been found to be deficient in mutant ventricles and sarcomere formation can be rescued by the addition of TM protein or cDNA. Although once thought to be due to endoderm induction our findings indicate a translational regulatory mechanism that may ultimately control the level of tropomyosin protein in axolotl hearts.

## Introduction

The Mexican axolotl, a facultative neotenous salamander, provides a valuable model to study heart development due to a cardiac lethal mutation (gene c) that affects only heart muscle [1, 2]. It has also been used extensively for organ regeneration research, particularly of its limbs and tails [3] but have included initial studies into the regeneration of the heart [4]. The axolotl cardiac gene c mutation is a Mendelian, autosomal recessive lethal mutation with significant effects on tropomyosin protein levels in the cardiac tissue. Morphological studies of the abnormal cardiomyogenesis in mutants have shown they lack organized myofibrils, have large collections of amorphous material, but still retain normal electrophysiological properties [5–7]. The embryos can survive for up to a fortnight post-hatching which is ideal for studying this process before the lack of circulation, secondary to abnormal sarcomere formation is lethal. The mutant axolotl heart provides a unique opportunity for studying the intricate process of cardiac development and for examining the specific functional role of each tropomyosin (TM) isoform in this process. Ultimately the protein level of TM is profoundly diminished in the ventricle of c/c mutant hearts, resulting in an absence of organized myofibrils and subsequently the inability to beat [5–8]. It is important to note that the conus is not deficient in tropomyosin protein, retains organized myofibrils and is capable of beating independently, unlike the atria and ventricle in mutant hearts [8]. Notably, the mutant hearts can be rescued *in situ* by supplying exogenous TM protein or TM cDNA in an expression construct under the control of an appropriate promoter(s) [9, 10]. Mutant hearts can also be rescued *in situ* by a specific non-coding RNA that is unrelated to TM [11–13]. However, the exact mechanism by which this RNA modulates the expression of tropomyosin is yet to be elucidated. To better understand the mechanism(s) effecting tropomyosin expression in mutant hearts, we undertook an extensive molecular characterization of the various isoforms of tropomyosin in the Mexican axolotl.

### Isoform diversity of tropomyosin in vertebrates

The thin filaments of striated muscle in vertebrate consist of actin, tropomyosin, the troponin (Tn) complex (Tn-I, Tn-C and Tn-T), tropomodulin, and a few other proteins [14]. Actin filaments interaction with Ca +2 governs muscle contraction and relaxation. Tropomyosin is a coiled coil actin-binding protein found along the length of seven actin monomers. A set of four known genes (*TPM1, TPM2, TPM3* and *TPM4*) that encode tropomyosin in vertebrates [15–19] give rise to various tropomyosin protein isoforms that play important roles in striated, smooth and non-muscle cells. There remains even further diversity in other models such as zebrafish where six tropomyosin genes have been identified [15]. The creation of different tropomyosin isoforms occurs through various mechanisms, including the use of different promoters, alternative mRNA splicing, different 3’ end mRNA processing and tissue specific translation control [20].

**Figure 1:**
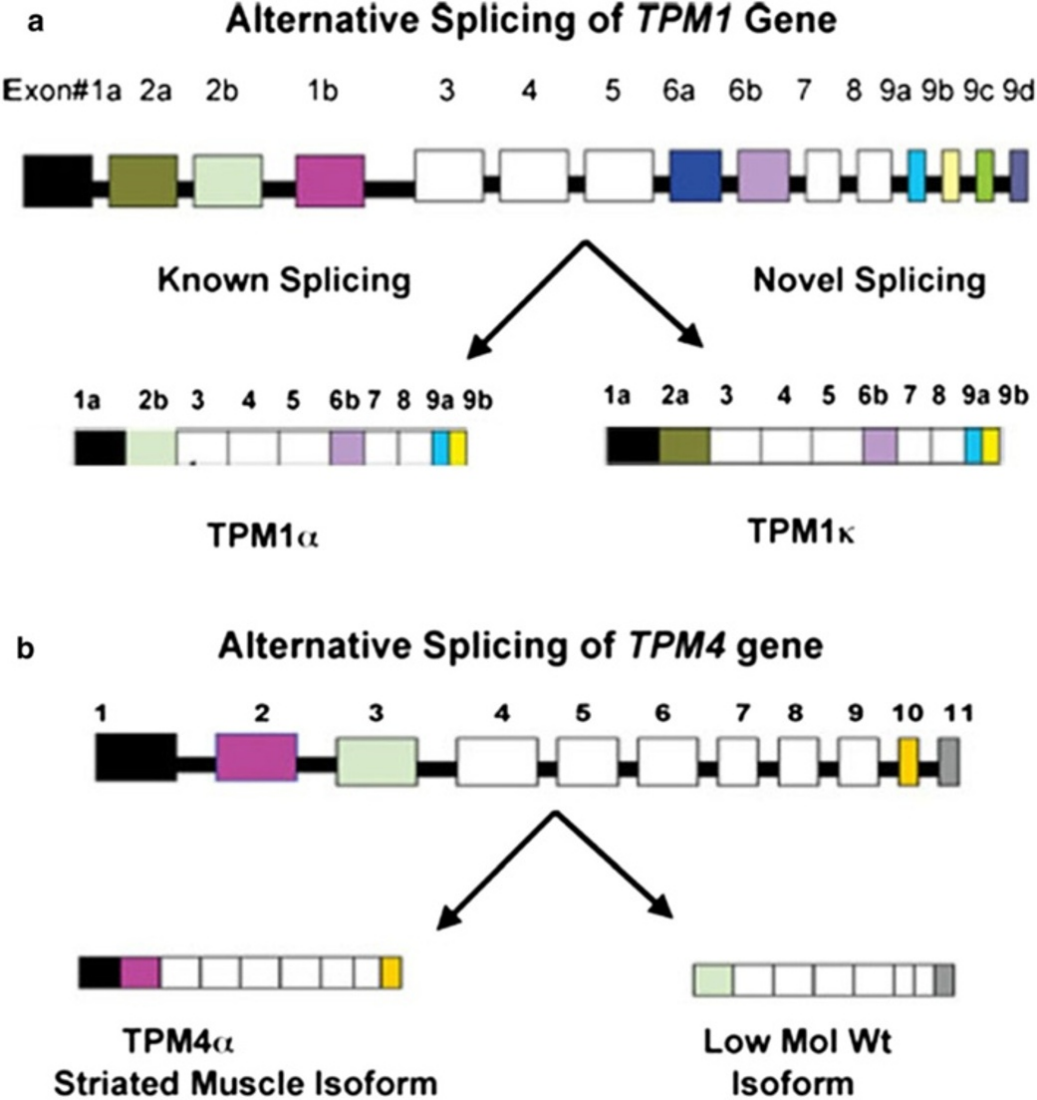
**Alternative splicing patterns of** ***TPM1*** **and** ***TPM4*** **genes. (a)** Exon composition of the *TPM1* gene and alternative splicing that generates two striated muscle specific isoforms -TPM1α and TPM1κ [26]. **(b)** Exon composition of *TPM4* gene (adapted from avian species as proposed by Fleenor et al. [30] and alternative splicing generate two isoforms in axolotl, TPM4α and a low-molecular weight TPM4 transcript.

We have cloned and sequenced the cDNA of three sarcomeric TM isoforms from cardiac tissues. These isoforms are designated as TPM1α, TPM1κ, and TPM4α [21–23] (Table 1). TPM1α, one of nine alternatively spliced isoforms of the *TPM1* gene, is known to be the major sarcomeric isoform in mammalian hearts [15–19]. We first identified and characterized another alternatively spliced sarcomeric isoform of the *TPM1* gene in axolotl hearts [22], designated TPM1κ. TPM1α and TPM1κ have an identical exon composition except for exon 2 where TPM1κ contains exon 2a instead of exon 2b (Figure 1 and Table 1). Exon 2a is characteristic of the smooth muscle type isoform (TPM1β) of the TPM1 gene (Figure 1 and Table 1). TPM1κ transcripts and its corresponding protein are expressed in both axolotl hearts and skeletal muscle there appears to be a differential translation of the transcript. Using qRT-PCR the expression level of TPM1κ transcripts is higher than TPM1α (ratio α:κ is 0.32) in adult axolotl hearts although TPM1κ protein is less than 10% of the total sarcomeric TM, as determined by CH1 antibody [24]. The opposite is true in adult skeletal muscle where the level of TPM1κ transcript is significantly lower compared to TPM1α (ratio of α:κ > 13) but the level of TPM1κ protein constitutes ~30% of the total sarcomeric TM [24] Similarly, the levels of expression of TPM1α and TPM1κ transcripts in human hearts are comparable but the actual TPM1κ protein level is only ~5% of the total sarcomeric TM [25]. Comparatively, TPM1α protein constitutes ~90-95% of the total TM in human hearts [24, 25] while TPM1κ is not expressed in human skeletal muscle at all [26]. TPM1κ transcripts are also expressed in embryonic chicken heart but not in adult heart and skeletal muscle [27]. It remains unknown if the protein is expressed since human TPM1κ antibody may not cross-react with chicken TPM1κ protein [25]. This discrepancy between transcript and protein levels in the two isoforms in heart tissue suggests that TPM1κ transcripts may undergo translational repression.

**Table 1:** **Exon compostion of various high molecular weight TM isoforms with old & new nomenclature** [15, 17, 19, 28, 29]

Nomenclature of various isoforms of TM referred to in this article	TPM Gene encoding the Isoforms: New Nomenclature (Old Nomenclature)	Various isoforms of TM currently known as	Exon composition	Nomenclature used in previous publications on axolotl
**TPM1α**	TPM1(α–TM)	Striated Muscle	1a,2b,3,4,5.6b,7,8,9a/b	ATmC-1/α-Tm-1
**TPM1β**	TPM1(α-TM)	Smooth Muscle	1a,2a,3,4,5,6b,7,8,9d	Sm α-Tm
**TPM1γ**	TPM1(α-TM)	TM-2Fibroblast	1a,2b,3,4,5,6b,7,8,9d	
**TPM1δ**	TPM1(α-TM)	TM-3Fibroblast	1a,2b,3,4,5,6a,7,8,9d	
**TPM1ϵ**	TPM1(α-TM)	TM-5aFibroblast	1b,3,4,5,6b,7,8,9d	
**TPM1κ**	TPM1(α-TM)	Novel Striated/Card	1a,2a,3,4,5,6b,7,8,9a/b	ATmC-2/α-Tm-2
**TPM2α**	TPM2(β-TM)	Striated/Sk Muscle	1a,2b,3,4,5,6b,7,8,9a/b	
**TPM3α**	TPM3(hTMnm)	Sk.Muscle	1a,2b,3,4,5,6b,7,8,9a/b	
**TPM4α**	TPM4(TM4)	StrTM4	1a,2b,3,4.5,6b,7,8,9a/b	ATmC-3/Str.TM-4

Accounting for the other tropomyosin genes other than TPM1, TPM2α (sarcomeric isoform of the *TPM2* gene) is also expressed in mammalian hearts in addition to the previously described TPM1α and TPM1κ The sarcomeric isoform of the *TPM3* gene, TPM3α, is only expressed in slow-twitch skeletal muscle. No sarcomeric isoform of the *TPM4* gene is expressed in mammalian striated muscles because the *TPM4* gene is truncated in mammals [15–17]. On the contrary, TPM4α is a major TM isoform in amphibian cardiac tissues [23, 31] and is the only isoform for sarcomeric TM in adult avian hearts [27, 30, 32].

### Sarcomeric tm protein in cardiac mutant axolotl hearts

**Figure 2:**
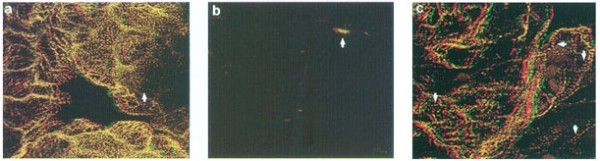
**Sarcomeric tropomyosin expression in normal, mutant, and TPM1α -transfected mutant hearts. a**. Confocal micrograph of stage-39 normal hearts stained with CH1 anti-tropomyosin antibdody (and rabbit anti-mouse lissamine rhodamine secondary antibody), well-organized sarcomeric myofibrils can be seen throughout the ventricle of the heart (arrow). **b**. Heart from stage-39 mutant embryo stained with CH1 does not show any organized myofibril, Only small areas of amorphous staining can be visualized within the ventricle (arrow). **c**. Stage-36 mutant heart lipofected with an expression construct containing a murine TPM1α cDNA under the control of α-Myosin Heavy Chain promoter, which subsequently induced TM and promoted myofibrillogenesis. Mutant heart stained with α-actinin primary antibody. Well-organized sarcomeric myofibrils can be seen throughout the heart (arrows). Staining of the Z-lines confirmed the sarcomeric organization seen in TPM1α transfected hearts that were stained with tropomyosin primary antibody (results not shown here) [ref]. Interestingly, statge-36 mutant heart tansfected with a murine TPM2α cDNA under the same promoter did express some TM protein but sarcomeric myofibrils did not form throughout the heart in contrast to TPM1α transfected hearts [7].

Among the various myofibril proteins, tropomyosin has been shown by a variety of experiments to be drastically reduced in cardiac mutant hearts [2, 6, 9, 12]. Increasing the intracellular levels of TM in cardiac mutant heart cells via introduction of FITC-labeled exogenous TM protein by itself or an expression construct allowing *in-vivo* TM production subsequently promoted myofibrillogenesis (Figure 2) [9]. Control mutant heart stained with CH1 monoclonal antibody specific for sarcomeric TM, demonstrated minimal staining when examined by confocal microscopy (Figure 2b). However, examination of mutant heart transfected with an expression construct of murine TPM1α cDNA under the control of mouse α-MYHC promoter demonstrated the formation of organized myofibrils (Figure 2c). The results prove mutant hearts are capable of forming cardiac myofibrils when provided with sufficient levels of tropomyosin protein. This unequivocally demonstrated the functional defect in the gene “c” mutation is the deficiency in tropomyosin protein although the underlying cause of this functional deficit is less clear. Interestingly, other myofibril structural proteins such as actin, myosin and myosin binding protein C (MyBp-C) were found to be at or near normal levels in the mutant hearts [5, 28] while one protein, tropomodulin which is intricately related functionally with sarcomere maturation is increased [33].

**Figure 3:**
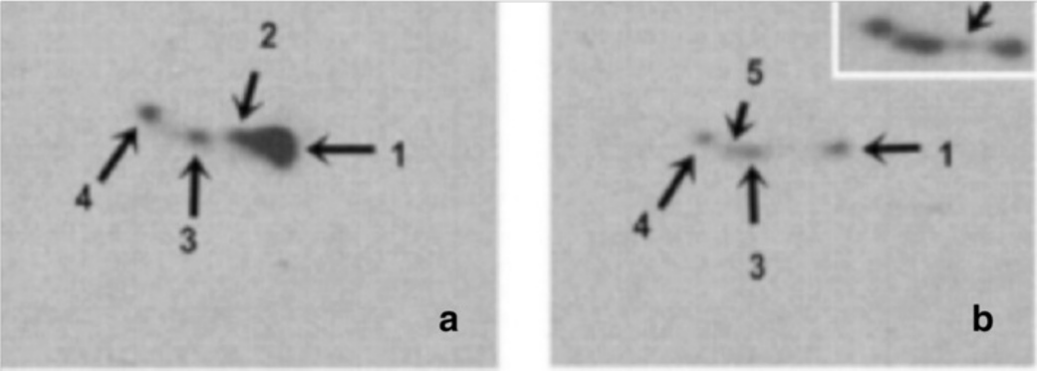
**2-D Western blot analysis of normal and mutant axolotl hearts using CH1 monoclonal antibody. a**. Stage-36 normal hearts show 4 different CH1-recognizable TM isoforms #1, #2, #3, and #4)**. b**. Mutant heart at stage-36 all show 4 CH1-recognizable TM isoforms as in normal hearts at much lower levels along with an extra isoform (#5). #5 isoform is detectable in normal hearts at later developmental stages (results not shown). The top right corner represents an overexposed blot B [12]. The figure was adapted from Zhang et al. [12].

The most comprehensive study on the analysis of sarcomeric tropomyosin protein expression in normal and mutant axolotl hearts were reported by Zhang et al. [12]. To determine whether multiple isoforms of tropomyosin exist in embryonic axolotl hearts and to verify if they are differentially regulated in mutant hearts, 2D western blot with the monoclonal antibody (CH1), was performed. Five different protein spots (tropomyosin isoforms) from both normal and mutant embryonic hearts at stages 36 to 42 were detected (Figure 3). All isoforms of tropomyosin detected by the sarcomer specific CH1 antibody (30) were located between pI 4 to 5 with molecular weight of ~38 kD. The results showed protein levels of the 4 CH1 recognized TM isoforms were decreased significantly in mutant hearts compared to normal hearts (Figure 3a and 3b) [12].

Although mutant axolotl hearts are deficient in sarcomere specific TM proteins, mRNA levels of each of three striated muscle isoforms (TPM1α, TPM1κ, and TPM4α) are comparable in normal and mutant hearts [12, 23]. Hence, the tropomyosin deficiency in mutant heart is not due to an insufficiency in transcription or post-transcriptional splicing [23]. We cloned and sequenced cDNAs of three isoforms from mutant hearts; no mutation(s) was detected in any of these cDNAs that may cause truncated non-functional TM isoform(s). Additionally, we have cloned and sequenced the promoter region of the *TPM4* gene from the DNA isolated from normal and mutant axolotl hearts and again, no differences were observed [34]. Hence, the possibility of insufficient transcription of the cardiac specific TPM4α isoform is highly unlikely. The most plausible explanation based on available evidence of TM deficiency in mutant hearts is a translational insufficiency of the tropomyosin transcripts in mutant hearts [12].

### Molecular analysis and manipulation of tropomyosin isoforms in normal and mutant axolotl hearts

As stated earlier, there are at least three striated muscle isoforms of tropomyosin present in the axolotl. Two isoforms of tropomyosin cDNA have been identified which apparently are derived from the single alpha-tropomyosin gene (*TPM1*) through alternative splicing [21, 22]. Spinner et al. [23] cloned another tropomyosin cDNA, which is the product of a TM4 type tropomyosin gene from axolotl heart. An expression construct with each of these isoforms upon transfection into mutant hearts canaugment tropomyosin proteinlevels and promotes myofibrillogenesis. The important question is whether or not any one of these isoforms alone and/or in various combination(s) is necessary for myofibrllogenesis in axolotl hearts *in vivo*. In order to address this issue we developed procedures for disruption of myofibrils in normal axolotl hearts mimicking the mutant hearts by lipofecting antibodies against sarcomeric TM into normal hearts *in situ*. Myofibrils in lipofected normal hearts indeed became greatly disorganized [10, 35]. As CH1 antibodies react with all three sarcomeric tropomyosins, it is not possible to evaluate the requirement of a particular isoform that is involved in cardiac myofibrillogenesis. Later we developed antibody against TPM1κ in rabbits using a 15-mer peptide sequence (LDELHKSEESLLTAD) derived from axolotl exon 2a [36]. Recall, TPM1κ is unique as a sarcomeric TM in that it contains exon 2a instead of exon 2b which is found in TPM1α. The affinity purified anti-TPM1κ antibody upon transfection could disarray the organized myofibrils in axolotl hearts [37]. The results strongly suggest that TPM1κ plays a critical role in myofibril formation in axolotl hearts.

**Figure 4:**
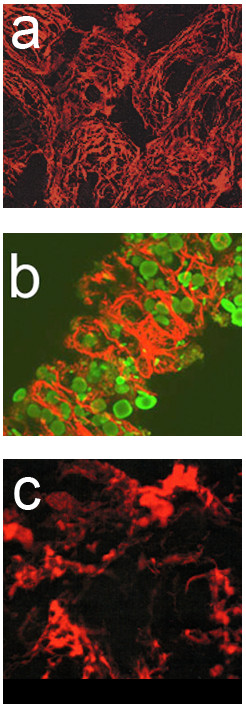
**Transfection of isoform-specific sense and anti-sense oligonucleotides in the ventricle of normal hearts. a.** Confocal microscopy of normal axolotl hearts transfected with TPM1a anti-sense oligonucleoted and subsequently stained with CH1 monoclonal antibody. TPM1a anti-sense oligonucleotide did not result in a drastic disruption of organized myofibrils in the ventricle, which is comparable with the normal untreated control hearts (figure not shown). Sarcomeric TM can be seen in most of the cells. Contractility of the anti-sense treated hearts were not affected. **b.** TPM1κ anti-sense transfection disrupted the myofibril organization in normal axolotl heart compared to TPM1κ sense transfection. Very little organized structure is seen when examined by the tropomyosin staining. The secondary antibody is contained within amorphous areas in the cells. **c**. It shows that the TPM1κ sense oligonucleoides did not affect the structure. Since no effect on myofibril structure with TPM1κ sense was observed, we tagged the oligonucleotide with FITC (green) to verify its presence and found it to be within the myocytes. Double staining of the nucleus (green) and the myofibrils at the periphery of the cells (red) can be seen. The green staining is ovoid in shape and primarily located at the center of the cells [10]. Confocal z-series images of stage ~38 embryonic axolotl hearts transfected with either TPM1κ anti-sense or sense oligonucleotides. Immunodetection of sarcomeric tropomyosin using CH1 monoclonal antibody is shown in red. The results depicted by this figure suggests that TPM1κ plays a critical role in maintaining the myofibrillar structure in embryonic axolotl hearts.

Additionally, isoform specific sense and anti-sense oligonucleotide was transfected into normal axolotl hearts. TPM1κ expression was blocked in whole embryonic axolotl heart by transfection of exon 2a-specific anti-sense oligonucleotide (Figure 4b). In contrast, myofibrils were unaffected in normal control heart when transfected with FITC label sense oligonucleotides (Figure 4c). RNA was isolated from treated and untreated hearts and subsequently RT-PCR was carried out with isoform specific primer-pairs. The results confirmed the lower transcript expression of TPM1κ in anti-sense treated hearts. The conclusion was substantiated by the *in vitro* analysis of the specificity of the TPM1κ anti-sense oligonucleotides used in this study. Confocal analysis of the sense and anti-sense oligonucleotide transfected normal axolotl hearts was carried out after staining with anti-tropomyosin antibody (CH1). Immunohistochemical analysis unequivocally confirmed that the inhibition of the expression of TPM1κ disrupted myofibril structure of the myofibrils in anti-sense transfected normal axolotl hearts. In contrast, TPM1α anti-sense oligonucleotide did not cause a disruption of the myofibrillar organization in axolotl hearts (Figure 4a).

**Figure 5:**
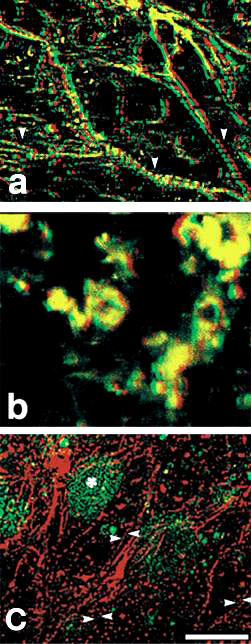
**Effect of transfection of TM isoform-specific sense- and anti-sense oligonucleotides on myofibril organization in hearts from normal axolotl hearts. a.** Stereo anaglyph of a 24 section confocal laser scanning microscope z-series of normal axolotl heart stained with CH1 anti-tropomyosin antibody. This type of image is shown because it demonstrates branching myofibrils in whole hearts. **b.** Stereo anaglyph of normal hearts transfected with TPM4α exon 2- specific anti-sense, 5′-T*A*C*T*AGCTCGTCCTCAAGC*T*G*C*-3′, where N* represents the phophothioate blocked oligonucleotide. Myofibirl organization was disrupted in a majority of the cardiomyocytes. Most of the tropomyosin appears to be in amorphous areas when expressed. Some of the cells do not appear to have a detectable level of tropomyosin. Gross morphology was normal and the cells appeared to be intact although myofibril structure was largely disrupted. The contractility was diminished significantly [36]. **c.** Normal heart transfected with TPM4α exon 2-specific sense chimeric olighonucleotide, 5′-fG*C*A*GCTTGAAGGCGAGCTA*G*T*A*-3, where *N represents phosphothioate blocked nucleotide, and fG represents G tagged with Fluorescein at the 5′end. This image is a compressed z-series of 2 sections that was not stereo offset but used to demonstrate double staining. Isolated pieces of myofibrils that were contained within these sections of the cardiac cells were stained with tropomyosin antibody (re, arrowheads). This image is primarily useful for demonstrating the presence green fluorescence (GFP) within the nuclear area of the cariomyocytes by five days (asterik). Hoescht staining of the nuclei in the same heart coincided with the FITC staining that were localized in a majority of nuclei (figure not shown). The figure was adapted from Spinner et al. (2004) [36].

In a separate study, we found that the antisense TPM4α oligonucleotide disrupted myofibril formation and inhibited beating in normal axolotl hearts, while the sense strands did not. A fluorescein-tagged sense oligonucleotide clearly showed that the oligonucleotide was introduced within the cells of intact hearts. The results implicate the essential role of TPM4α in cardiac myofibrillogenesis (Figure 5) [8, 10, 36].

### Differential expression of tropomyosin in conus and the ventricle

**Figure 6:**
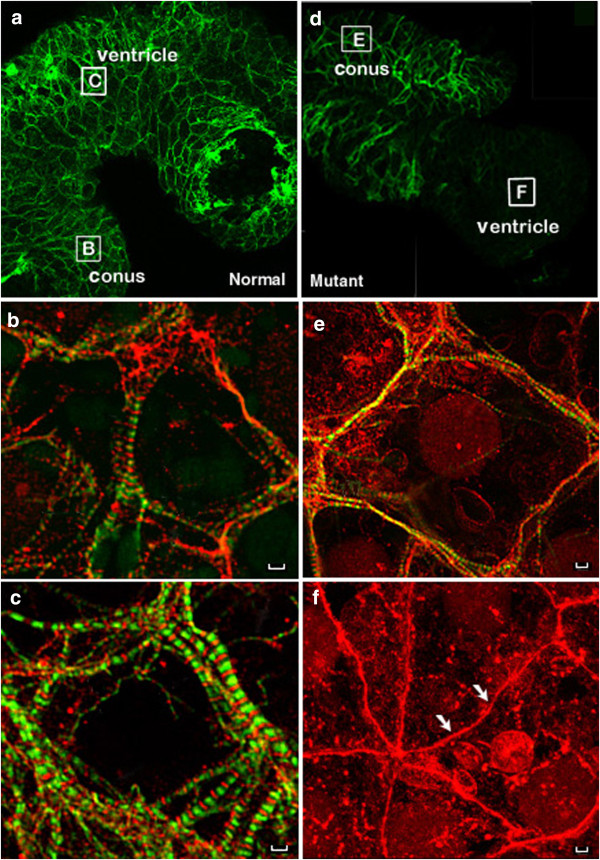
**Tropmodulin but not tropomyosin found in cardiac “c” mutant axolotl ventricle.** CH1 anti-sarcomeric TM antibody labels the conus but not the ventricle in stage 38/39 cardiac mutant axolotl hearts. Stage 38/39 normal **(a**–**c)** and mutant **(d**–**f)** axolotl hearts were double stained for immunofluorescent microscopy with CH1 anti-sarcomeric TM antibody (FITC channel) and polyclonal anti-tropomodulin antibody, R1749 (Rhodamine channel). Only the FITC channel is shown for whole heart images **(a, d)** demonstrating the lack of staining in the ventricle of mutant hearts while all higher magnification images utilize a dual rhodamine-FITC filter. Letter labeled boxes in A and D correspond with the area shown in their respective higher magnification image. TM labeled with CH1 antibody is found in a cross-striated pattern in the ventricle of normal hearts **(c)** and in the conus of both normal **(b)** and mutant **(e)** hearts. Tropomyosin staining is markedly absent in mutant ventricle **(f)** resulting in a failure of normal sarcomere formation with tropmodulin found in linear arrays near the surface of the membranes (Arrows, F). Scale Bar = 100 μm.

Despite the dramatic and lethal effects of the homozygous cardiac gene “c” mutation in the axolotl ventricle, the conus of the heart beats and has organized myofibrils (Figure 6). In order to understand whether various TM isoforms are differentially expressed in different segments of the heart and whether the known TM isoforms contribute to myofibril formation in a segment specific manner, we employed anti-sense oligonucleotides to separately knockdown post-transcriptional expression of TPM1α and TPM4α in axolotl heart segments. We evaluated the organization of myofibrils in the conus and ventricle of normal and *cardiac* mutant hearts using immunohistochemical techniques. We concluded that the TPM1α isoform, a product of the *TPM1* gene, was essential for myofibrillogenesis in the conus, whereas TPM4α, the striated muscle isoform of the *TPM4* gene, was essential for myofibrillogenesis in the ventricle. Our results support the segmental theory of vertebrate heart development and suggest the conus is a different transcriptional tissue unit. Since the conus is an outflow tract structure and in humans is a conical pouch of the right ventricle from which the pulmonary artery arises it will be interesting to examine tropomyosin isoform diversity in these two segments in other systems including humans [8]. Development of the conus appears to be unaffected in the mutant heart and is comparable to the normal heart. The different functions of the heart segments would suggest that different isoforms could be needed in accordance with that function. The physiologic characteristics necessary for the ventricle versus the conus and subsequent outflow tract are different [38]. Isoform diversity in specific heart segments includes tropomyosin but also could include other sarcomeric proteins such as myosin heavy chain [39] Further studies could examine the localization and function of specific isoforms in adult heart segments. There has also been research on isoform diversity and the relationship to diseases such as dilated cardiomyopathy [25]. These studies suggest that with changing physiologic parameters, the isoforms can also be changed. Ultimately, the study of segment specific tropomyosin isoforms may help in the understanding of time and function specific sarcomeric proteins and their relationship to regeneration of heart function in damaged hearts.

### Promotion of myofibrillogenesis in mutant hearts *in situ*by a non-coding rna

A noncoding RNA, Myofibril-Inducing RNA (MIR) is capable of promoting myofibrillogenesis and heart beating in the mutant (c/c) axolotl hearts *in situ*[11]. Zhang et al. [12] demonstrated that the *MIR* gene is essential for tropomyosin (TM) expression in axolotl hearts during development at the level of translation or post-translation. qRT-PCR using isoform-specific primer-pairs showed that mRNA expression of three sarcomeric tropomyosin isoforms (TPM1α, TPM1κ, and TPM4α) in untreated mutant hearts and in normal hearts knocked down with double-stranded MIR (dsMIR) are similar to untreated normal. However, at the protein level, sarcomeric tropomyosin isoforms detected with CH1 monoclonal antibodies, are significantly reduced in mutant and dsMIR treated normal hearts. However, this study neither showed the mechanism by which MIR may induce sarcomeric TM synthesis in axolotl hearts nor addressed the role of specific tropomyosin isoforms in cardiac myofibrillogenesis.

Recently, Kochegarovr et al. [40] randomly cloned RNAs from fetal human heart. RNA from one of the clones (clone #291) was found to promote myofibril formation in mutant axolotl *in situ*. This RNA induced expression of cardiac markers in mutant hearts: tropomyosin, troponin and α-syntrophin. The nucleotide sequences of the cloned RNA matches in partial with the human microRNA-499a and b, although it differs in length. qRT-PCR data suggest this RNA may induce the TPM4α (ATmC-3) isoform in mutant heart, producing more sarcomeric tropomyosin protein and subsequently promote myofibril formation. The mechanism by which this non-coding RNA induces tropomyosin in mutant hearts may well be different from that of MIR, which acts at the post transcriptional level [12].

## Review and conclusions

Although our complete understanding of the mechanism of tropomyosin expression in mutant axolotl hearts as well as the nature and function of gene “c” is far from over, we would like to end this review with a positive note. Our finding of sarcomeric TM isoform TPM1κ in axolotl led to the discovery of this isoform in human hearts [26]. Unlike in axolotl, it is not expressed in human skeletal muscle. Most importantly, an upregulation of TPM1κ protein has been reported in hearts from human dilated cardiomyopathy patients [25]. However, it is not yet known whether the upregulation of TPM1κ is the cause or a consequence of cardiomyopathy in this patient. Our anti-sense experiments suggest strongly the functional significance of TPM1κ in axolotl hearts. In addition, the lower expression level of TPM1κ protein in axolotl heart and skeletal muscle [24] and also in human hearts [25] points towards translational repression of TPM1κ. Further, upon injection intraperitoneally into juvenile axolotl, Shz-1, a cardiogenic small molecule, augmented the expression levels of transcripts of TPM1α, TPM1κ, and TPM4α in hearts. But the increased transcript level did not resulted into increased sarcomeric TM protein expression [41]. Finally, although the transcript levels of all TPM isoforms in normal and mutant axolotl heart ventricles are comparable, the proteins of all three isoforms are diminished significantly. This observation also point towards the translational repression of TM in cardiac tissues [42]. The evidence for translational control of sarcomeric TM in mammalian hearts was originally came from the works from the laboratory of Dr. David Wieczorek, University of Cincinnati, Cincinnati, OH [29, 43]. Rethinesamy et al. [29] and Blanchard et al. [44] independently ablated one of the two alleles of the *TPM1* gene in mice that resulted in half of the TPM1α transcripts in ablated mice hearts compared to wild-type. However, TPM1α protein level was unchanged in ablated mice hearts suggesting a higher translational efficiency of TPM1α transcripts in ablated mice hearts. Again, Rajan et al. [25] reported that although the level of transcripts of TPM1α and TPM1κ human hearts is parallel, TPM1κ protein is only ~5% of the total sarcomeric TM whereas TPM1α protein constitutes about 90-95% of the total sarcomeric TM. The results strongly suggest the translational repression of TPM1κ transcripts in human hearts. The immediate future goal of our laboratory is to explore further the translational repression of tropomyosin expression in vertebrate hearts as well as to find out the functional role of TPM1κ.
